# The Effectiveness of Price Promotions in Purchasing Affordable Luxury Products: An Event-Related Potential Study

**DOI:** 10.3389/fnins.2022.818503

**Published:** 2022-02-23

**Authors:** Kunpeng Jing, Lele Chen, Yupeng Mei

**Affiliations:** School of Economics and Management, Yanshan University, Qinhuangdao, China

**Keywords:** affordable luxuries, price promotions, event-related potentials, N2, LPP, consumer neuroscience

## Abstract

Similar to traditional luxuries, affordable luxuries enjoy a high level of perceived product quality and perceived social status, but the effectiveness of price promotions of purchasing affordable luxury products is different from that of traditional luxuries. In order to further investigate the purchases of affordable luxuries, we used event-related potential (ERP) technology to reveal the formation of the purchase intention toward affordable luxuries at original prices (high or low) and current prices (discounted or non-discounted). Compared with the high-priced affordable luxury without a price promotion and the low-priced affordable luxury with a price promotion, consumers showed a stronger intention toward the high-priced affordable luxury with a price promotion, by weighing up three factors, perceived product quality, perceived social status, and perceived monetary saving at the behavioral level. A shorter reaction time emerged in the price promotion condition than in the absent price promotion condition when the original price was low. At the neural level, a decrease in N2 amplitude was found in the high original price and discounted current price condition than the high original price and non-discounted current price condition and the low original price and discounted current price condition, respectively, suggesting that the price information of the latter two conditions might not be the expectation information of subjects, and thus, the enhanced conflict is produced. The high-priced affordable luxury product without a price discount evoked a more positive LPP amplitude than the high-priced affordable luxury product with price promotions or than the low-priced affordable luxury item without price promotions, demonstrating that participants could regard the former as an evaluative inconsistent condition and the latter as evaluative consistent conditions. These results are helpful to better understand the effects of price promotions on purchasing affordable luxury products at different original prices.

## Introduction

Traditional luxuries are products of high quality and high price, and it makes consumers feel socially superior (Mazzocco et al., 2012) and proud (Bellezza and Keinan, 2014). Traditional luxuries tend to combine high perceived prestige with extremely high price premiums. In contrast, affordable luxury products are sold at reasonable price premiums for mass and still enjoy a neutral and reasonable level of perceived prestige (Silverstein and Fiske, 2005). Thus, affordable luxuries are more accessible for middle-class or lower-class consumers (Truong et al., 2009).

There are some similar findings for traditional and affordable luxury consumption. Compared with necessities, consumers think that affordable luxuries representing a signal of economic power have the same goods characteristics (such as high quality, high priced, and exclusive) as the traditional luxuries (Mundel et al., 2017). Not only for traditional luxuries but also for affordable luxuries, the self-monitoring and need for uniqueness can predict a consumer’s attitude toward a brand (Ajitha and Sivakumar, 2019): self-monitoring in association with social identity can positively affect social-adjustive attitude toward a brand (Shavitt et al., 1992) and need for uniqueness involving self-image and social representation can also positively affect value-expressive attitude (Vigneron and Johnson, 2004). High-order social and psychological desires, as one of the most influential factors, can drive both traditional (Wiedmann et al., 2009) and affordable luxury purchases (Shahid et al., 2021).

On the other hand, there is a potential difference between traditional and affordable luxuries in terms of the influence of price promotions. Price promotion as one of marketing promotions is often employed by companies and can increase perceived monetary savings (e.g., Chandon et al., 2000). However, a price promotion sometimes cannot spur the demand for goods. People who are high in need for status exhibit a negative attitude toward a luxury hotel and have less intention of returning to a hotel when a price promotion is presented, but it is not the case in consumers who are low in need for status (Yang et al., 2015). The reason for the results is that the former group’s people regard price exclusivity for luxury hotel as a means of signaling his or her wealthy identity (Han et al., 2010) but the price promotion can make a luxury hotel more accessible to the masses. In contrast, offering a price discount for, respectively, a low-priced room in a luxury hotel can increase booking behavior (Jang and Moutinho, 2019). The price of this type of room is more reasonable for the masses. Consumers enjoy a reasonable level of perceived social status, and they place a greater weight on the benefit of luxury accommodation and amenities. As mentioned above, the main difference between traditional and affordable luxuries is price. Therefore, affordable luxury products are similar to a low-priced room in a luxury hotel, and price promotions have a positive influence in promoting the purchases of affordable luxury. Additionally, Jang and Moutinho (2019) showed that the effectiveness of price promotions in booking behavior depends on the extent to which consumers perceive social status for luxuries. Only when perceived social status is high is price promotion effective. Considering that perceived social status is positively related to the price of affordable luxuries (Truong et al., 2009), when the original price of affordable luxuries is high, the need for social status could be met first, and price promotions could increase perceived monetary savings to promote purchases; when the original price is low, perceived social status is also low, and thus, even if price promotions mean monetary savings, the savings could not influence purchase intention. In sum, the higher the original price of affordable luxuries, the more effective the price promotion is, and the moderation effect could be mediated by perceived social status.

To further investigate these effects, we have had insight into neural mechanisms underlying behaviors on affordable luxury items. Specifically, two aspects are considered: how price promotion information is processed for high-priced and low-priced items, respectively, and what ultimately determines consumers’ final purchase decision.

Event-related potentials (ERPs) as a noninvasive scalpoimaging technique was applied to provide information about the timing of brain activities in the present study. The ERP technique can measure a participant’s response effectively and objectively, and it is much more inexpensive than the other neuroscience techniques, such as functional magnetic resonance imaging (fMRI), position emission topography (PET), and event-related magnetic fields (ERMFs), thus, more accessible to many researchers. ERPs can provide a window into consumer behaviors and is helpful for marketing researchers and professionals.

Two ERP components, N2 and late positive potential (LPP) were considered in the study. N2 is a negative-going ERP component, peaking between 200 and 400 ms post-stimuli. The component is related to cognitive conflict (Folstein and Van Petten, 2008). For example, in the go/no-go paradigm, participants have to respond to one stimulus (go response) and do not to the other stimulus (no-go response). When the no-go stimulus is presented, this produces conflict between go response and no-go response and leads to an increase in N2 amplitude (Bruin and Wijers, 2002). More recent work about consumer neuroscience showed that although consumers show a preference for hedonic products with mixed promotion (a combination of price discount and donation to charity), they anticipate more negative pure price promotion to reject purchases (Mei et al., 2021). Thus, when the positive promotion information is presented, conflict and a higher N2 amplitude emerge. Actually, discrepancy for N2 amplitude between conditions depends on decision pattern. Subjects tended to purchase high-quality products, leading to conflict for the product information with risk of low quality (Wang et al., 2016; Jin et al., 2017). Participants wanted to reject purchases, producing anticipation conflict in the more positive mixed promotion (Mei et al., 2021). In the go/no-go paradigm, subjects wanted to make a manual response, and thus, a higher N2 amplitude was elicited in the no-go condition (Bruin and Wijers, 2002). As mentioned above, consumers could anticipate that price promotions emerge in high-priced affordable luxuries rather than in low-priced ones. Therefore, when the original price is high, a non-discounted current price could produce conflict and leads to an increase in N2 amplitude compared with a discounted price; when the original price is low, there could be no difference between the price promotion condition and the absent price promotion condition in N2 amplitude.

Late positive potential (LPP), a member of the P300 family, is a positive component that peaks approximately 600 ms post-stimuli. A body of research demonstrates that this ERP component is related to the evaluative properties of stimuli. For example, both positively and negatively affective stimuli can elicit an enhanced LPP amplitude compared with neural stimuli (Hajcak and Olvet, 2008). During online purchase decision, participants categorized online reviews into consistent positive reviews, consistent negative reviews, and inconsistent reviews (Chen et al., 2010). Two types of consistent reviews as higher evaluation categorizations evoke a larger LPP amplitude than inconsistent reviews. Besides, this cognitive process of evaluative categorization is sensitive to a preceding context as well (e.g., Cacioppo et al., 1993; Ito et al., 1998). Using the oddball paradigm, Dhont et al. (2012) created the sequence counting one target within a context of some context stimuli. When participants were instructed to indicate the valence of stimuli (positive or negative), the valence category of target, which is inconsistent with one of context stimuli (e.g., positive target and negative context stimuli), led to an enhancement of LPP amplitude than the consistent target regardless of political target words and non-political ones (Dhont et al., 2012). In a sequential priming paradigm, subjects have to pay attention to the first stimuli (the prime) and indicate the pleasantness of the second stimuli (the target). A larger LPP can be observed for evaluatively incongruent stimuli (i.e., pleasant–unpleasant, unpleasant–pleasant) than evaluatively congruent stimuli (i.e., pleasant–pleasant, unpleasant–unpleasant) (Zhang et al., 2010; Herring et al., 2011). During the current experiment, the original price was presented first and then the current price was presented. High original price meaning high social status (Truong et al., 2009) serves as positive stimulus; meantime price promotions could be effective, suggesting that a non-discounted current price serves as a negative stimulus, and a discounted price serves as a positive stimulus. Therefore, when the original price is high, a non-discounted current price could produce inconsistent evaluation compared with a discounted current price and a non-discounted current price could lead to a larger LPP; low original price meaning low social status serves as positive stimulus; meantime price promotions could be ineffective, suggesting that both a non-discounted price and a discounted price serve as negative stimuli. Therefore, there could be no difference between the price promotion condition and the absent price promotion in LPP amplitude.

In summary, we predict that the original price of affordable luxuries could moderate the effectiveness of price promotions, and the effect could be mediated by perceived social status. We expect that the moderation effect of original price could be manifested in N2 and LPP amplitudes.

## Materials and Methods

### Participants

Twenty right-handed female graduate students (mean age: 24.10, S.D. = 1.12) who were familiar with the preselected luxury brand and had purchased the luxury brand products were recruited. The brand is targeted at women, and thus, we only recruited female participants. These subjects were all native Chinese speakers and had normal or corrected-to-normal vision with no history of neurological disorders or mental diseases. The study was approved by the institutional review board, and written informed consent was provided before the experiment. The data of one participant was discarded due to excessive artifacts.

### Experimental Stimuli

To avoid the effect of consumer preference for different items, only one Coach handbag with high sales was used from the official flagship store of Coach in Taobao, which is one of the largest online retailers in China. Coach is a popular affordable luxury brand around the world (Wang, 2013; Wang and Qiao, 2020). Two pictures including the front and the back of this handbag were created and processed to the size of 300 × 270 pixels. In total, there are 12 stores in this experiment and their names are represented by English letters from A to L (totaling 12). In Taobao, the prices of products in other stores are often lower than that in the official flagship store. Therefore, based on the original price (4,900 yuan, approximately 760 dollars) in the official flagship store, eight kinds of different prices, 4,820, 4,830, 4,840, 4,850, 4,860, 4,870, 4,880, and 4,890 yuan, in other stores were regarded as high price, and the other eight kinds of different prices, 2,820, 2,830, 2,840, 2,850, 2,860, 2,870, 2,880, and 2,890 yuan, were regarded as low price. To check the manipulation of this factor, a questionnaire was conducted before the experiment, and it consisted of two questions: to what extent do you agree that a Coach handbag with the price range of 4,820 to 4,890 is a high-priced item, and to what extent do you agree that a Coach handbag with the price range of 2,820 to 2,890 is a low-priced item (where 1 = “not at all agreeable” and 7 = “extremely agreeable”)? Confirming the manipulation, the ratings of two questions were larger than the midpoint of the questionnaire of four (*p*s < 0.05). In order to make a decision easily and quickly, the difference between the discounted price and the corresponding original price was 1,000 yuan according to offers from past shopping festivals. The stimuli contained 64 pairs of product pictures with the original and the present price information, i.e., 2 pictures × 16 categories of original price information (including eight kinds of high-priced information and eight kinds of low-priced information) × 2 price promotion conditions (present or absent). Each pair of stimuli was repeated three times, and thus, there were 192 trials in the entire experiment.

### Procedure

Participants sat in a comfortable chair in a sound-attenuated room 80 cm away from the 23-inch monitor (1,360 × 768 pixels, 60 Hz). A keyboard was provided for participants to input appropriate choices. The Psychophysics Toolbox (Brainard, 1997) was used to control the stimuli and to acquire the behavioral data.

Prior to the formal experiment, participants were informed to image that they had a stable job with a high income and tended to purchase one Coach handbag during the Double 11 shopping festival. They needed to indicate their purchase intention according to price information from different stores. Double 11 is one of the biggest shopping festivals in China. As such, participants were willing to trust that the discounted price in this festival is true.

**FIGURE 1:**
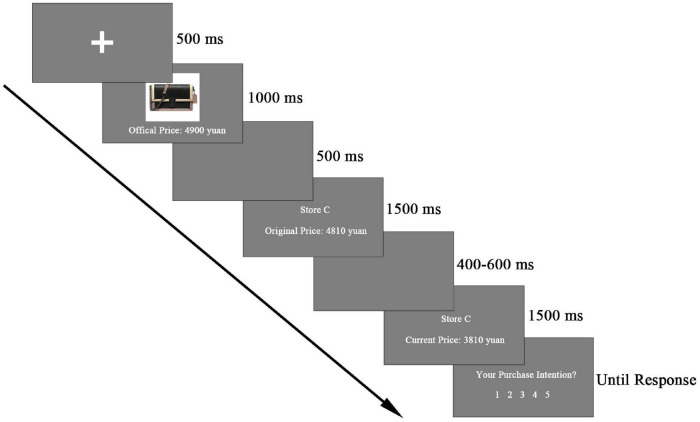
Sequence of stimuli in each trial. First, an affordable luxury product picture was presented. Then an original price (high or low) in a store was presented, and afterward the store’s current price (discounted or non-discounted) was presented. At last, participants need to give their purchase intention according to the original price and current price. Epochs were extracted after current price information onset.

Figure 1 illustrates the trial structure. Each trial began with a fixation cross presented for 500 ms. Following the fixation, an affordable luxury product picture along with the official price information was displayed against a gray background for 1,000 ms and was then replaced by an empty screen for 500 ms. The first presentation of official price aims to decrease individual difference. Specifically, if the official price subjects estimated were higher (lower) than the actual official price, they would probably serve all the original prices as low (high) price. Thus, to decrease this effect, the official price presented first in this experiment can provide the subjects with a reference to judge whether the original price was high or low in a clearer way. Afterward, the original price information was presented for 1,500 ms, with the phrase “Original Price” and the price placed right below the store name, followed by a blank screen ranging from 400 to 600 ms. Then the discounted price information was displayed for 1,500 ms. In this stimulus, the presentation was similar to the original price information stimulus. Finally, subjects have to rate the purchase intention for this offer in the store on a five-point scale (1 = I do not want to purchase the item with this offer at all, 5 = I really want to purchase the item with this offer). Each store name was randomly presented 16 times, and all trials were randomly classified into four blocks.

After the experiment, participants were instructed to complete a rating task. For different-priced affordable luxury products (high-priced vs. low-priced) with a price promotion or without, they have to, respectively, rate perceived monetary savings, perceived quality, and perceived social status. Perceived monetary savings was accessed with three items (α = 0.97): “I really save money by buying this coach handbag,” “I feel that I am getting a good idea by buying this coach handbag,” and “I really spend less by buying this coach handbag” from Chandon et al. (2000). Perceived quality was addressed with five items (α = 0.98): “The likelihood that this coach handbag would be reliable is very high,” “The workmanship of this coach handbag would be very high,” “This coach handbag should be of very good quality,” “The likelihood that this coach handbag is dependable is very high,” and “This coach handbag would seem to be durable” from Dodds et al. (1991). Perceived social status was accessed with three items (α = 0.97): “Buying this coach handbag means wealth,” “Buying this coach handbag is a social status symbol for me,” “Buying this coach handbag is a symbol of success and prestige,” and “Buying this coach handbag is to show off or to be noticed” from Correia et al. (2018).

### Electroencephalogram Recording and Analysis

Electroencephalogram (EEG) was recorded with a Brain actiCHamp amplifier (Brain Products GmbH, Munich, Germany) at 64 scalp sites using Ag/AgCl electrodes attached to an elastic cap. All scalp electrodes were referenced on-line to the Cz site. The electrodes placed supra- and intra-orbital to both eyes and lateral to the outer canthi of both eyes were used to measure the electrooculogram (EOG). Electrode impedances were kept below 10 kΩ. EEG signals were sampled at a rate of 500 Hz.

BrainVision Analyzer 2.1 (Brain Products) was used to preprocess the EEG signals. Off-line low-pass filter was 30 Hz (24 dB/Octave) and the average of left and right mastoid recording as an offline reference. EOG artifacts were corrected by the independent component analysis (ICA) method in the Analyzer program. The epoch was extracted from 200 ms before the onset of current price information to 800 ms after its onset. Epochs with a maximum difference between two adjacent voltage points > 75 μV were rejected. The EEG recordings for each subject were averaged for the four conditions (2 categories of original price × 2 categories of current price).

Based on the visual inspection of the grand average waveforms and the neuroscience research mentioned in the introduction, the 270- to 330-ms time window and 10 electrodes (F3, F1, Fz, F2, F4, FC3, FC1, FCz, FC2, and FC4) in the frontal-central area were specified for the N2 component, the 550- to 700-ms time window and 10 electrodes (CP3, CP1, CPz, CP2, CP4, P3, P1, Pz, P2, and P4) in the central-parietal area were specified for the LPP component. Repeated-measure analyses of variance (ANOVAs) were performed for behavioral data and ERP data. The Greenhouse–Geisser correction was used when necessary (uncorrected d*f* was reported with the ε and corrected *p*-value). All values of purchase intention, reaction time, mean scores of questionnaire, and ERP amplitudes are expressed as mean ± SEM.

## Results

### Behavioral Results

**FIGURE 2:**
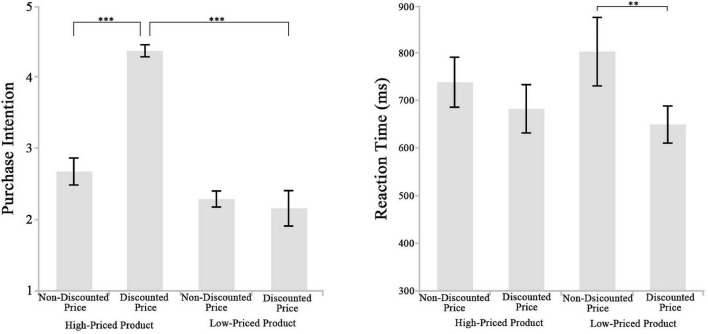
Mean purchase intentions **(left)** and mean reaction times **(right)** for four conditions (high-priced product with discounted price or with non-discounted price, low-priced product with discounted price or with non-discounted price). The error bars suggest standard error of the mean. **p < 0.01, ***p < 0.001.

Behavioral results are shown in Figure 2 and Tables 1–3. The purchase intention and reaction time were analyzed, respectively, by two-way 2 (original price: high vs. low) × 2 (price promotion: present vs. absent) repeated-measure ANOVAs. Regarding the purchase intention, two significant main effects of the original price [*F*_(1,19)_ = 31.804, *p* < 0.001, η${}_{\text{p}}^{2}$ = 0.626] and the price promotion [*F*_(1,19)_ = 18.959, *p* < 0.001, η${}_{\text{p}}^{2}$ = 0.499] and a significant interaction effect between these two factors [*F*_(1,19)_ = 44.894, *p* < 0.001, η${}_{\text{p}}^{2}$ = 0.703] were found. As a confirmatory follow-up, the simple effect analyses revealed that when the original price was high, participants showed stronger purchase intention to the affordable luxury product with a price promotion (4.353 ± 0.097) than that without (2.661 ± 0.203, *p* < 0.001) and that when the original price was low, there was no significant difference between the absent (2.273 ± 0.123) and present (2.144 ± 0.257, *p* = 0.599) price promotion conditions. As for the reaction time, there was a significant main effect of the price promotion [*F*_(1,19)_ = 12.067, *p* = 0.003, η${}_{\text{p}}^{2}$ = 0.388] and insignificant main effect of original price [*F*_(1,19)_ = 0.368, *p* = 0.551]. The interaction effect was marginally significant [*F*_(1,19)_ = 3.120, *p* = 0.093, η${}_{\text{p}}^{2}$ = 0.141]. The simple effect analyses showed that when the original price was high, significant difference between absent (737.069 ± 53.924 ms) and present (680.799 ± 52.622 ms, *p* = 0.140) price promotion conditions was not found and that when the original price was low, subjects responded to the discounted price (648.322 ± 40.522 ms) more quickly than the non-discounted price (801.621 ± 73.399ms, *p* = 0.003).

**TABLE 1:** The ANOVA results of the rating task.

	High-priced product		Low-priced product		Main effect of original price *F*(*p*)	Main effect of price promotion *F*(*p*)	Interaction effect *F*(*p*)
Price promotion	Present	Absent	Present	Absent			
Perceived monetary savings	3.42 ± 0.63	2.52 ± 0.81	6.30 ± 0.56	5.72 ± 0.60	342.295 (<0.001)	41.131 (<0.001)	2.660 (0.119)
Perceived quality	5.45 ± 0.82	5.95 ± 0.60	2.27 ± 0.74	2.89 ± 0.79	148.248 (<0.001)	45.003 (<0.001)	0.383 (0.543)
Perceived social status	3.86 ± 0.97	4.14 ± 0.80	2.13 ± 0.92	2.46 ± 0.75	44.126 (<0.001)	15.111 (0.001)	0.291 (0.596)

**TABLE 2:** The linear mixed models (LMM) analysis results for mediation effect of perceived social status.

Dependent variable	Independent variable	*b*	*t*	*p*-Value
Purchase intention	Price promotions	1.013	4.79	<0.001
	Original price	0.518	1.195	0.237
	Perceived monetary savings	–1.09	–3.194	0.004
	Original price × Perceived monetary savings	1.379	3.467	0.001
Perceived monetary savings	Price promotions	0.707	4.945	<0.001
	Original price	–3.015	–20.879	<0.001
Perceived social status	Price promotions	–5.253	–1.604	0.113
	Original price	1.397	9.015	<0.001
Perceived quality	Price promotions	–0.317	–3.381	0.001
	Original price	1.776	19.001	<0.001
Purchase intention	Price promotions	1.234	5.843	<0.001
	Original price	–0.294	–0.637	0.527
	Perceived monetary savings	–1.041	–2.16	0.042
	Perceived social status	–0.266	–1.862	0.071
	Perceived quality	0.685	3.256	0.003
	Original price × Perceived monetary savings	1.399	2.112	0.043
	Perceived social status × Perceived monetary savings	–0.312	–1.828	0.073
	Perceived quality × Perceived monetary savings	0.217	0.958	0.343

**TABLE 3:** The LMM analysis results for the groups of high perceived social status and low perceived social status.

	Independent variable	*b*	*t*	*p*-Value
High perceived social status	Price promotions	1.515	7.482	<0.001
	Perceived monetary savings	0.193	1.792	0.087
	Perceived quality	0.279	2.778	0.013
Low perceived social status	Price promotions	0.526	2.269	0.029
	Perceived monetary savings	−0.34	−1.882	0.07
	Perceived quality	0.245	1.416	0.169

As shown in Table 1, the mean score for each factor in the rating task was, respectively, collected in a 2 (original price) × 2 (price promotion) ANOVA. All the main effects were significant, whereas all interaction effects were insignificant. Perceived monetary savings in the low-priced product condition was higher than that in the high-priced product condition and there was an increase in the price promotion condition compared with the absent price promotion condition. Moreover, subjects perceived higher product quality and higher social status for both the high-priced affordable luxury product and the absent price promotion.

**FIGURE 3:**
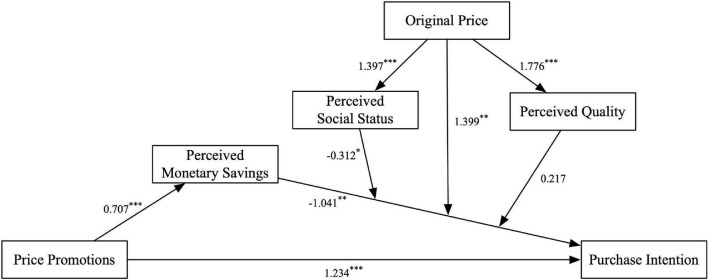
The casual relationship model among variables and the results of moderation and mediation analyses. The figures near arrows suggest coefficients of independent variables. *p < 0.1, ^**^p < 0.05, ^***^p < 0.001.

Furthermore, we used the linear mixed models (LMM) procedure in SPSS to show how the original price affects the effectiveness of price promotions. The LMM procedure extends the generalized linear model to allow for moderation and mediation analyses of repeated measurements (Kenny et al., 2003; Huang and Yuan, 2017). As mentioned in the *Introduction* section, we hypothesized that the influence of original price was mediated by perceived social status. To confirm this hypothesis, we took causal steps approach (Baron and Kenny, 1986; Huang and Yuan, 2017). Step 1: examining whether perceived social status can moderate the influence of perceived monetary savings on affordable luxury purchases. If the effect is significant, go to the next step. Step 2: examining whether the original price can influence perceived social status. If the effect is significant, go to the next step. Step 3: when considering the moderation effect of original price, examining whether perceived social status can moderate the influence of perceived monetary savings on affordable luxury purchases. If the effect is significant, the mediation effect of perceived social status would be significant. The results^1^ (Table 2) supported the interaction effect hypothesis. The main relationships among these variables are shown in Figure 3. Additionally, a median split (median = 3.25) was used to divide perceived social status into two groups for further analyses. As shown in Table 3, when consumers perceived high social status, the effect of perceived quality was significant (*b* = 0.279, *t* = 2.778, *p* = 0.013), and the effect of perceived monetary savings was marginally significant (*b* = 0.193, *t* = 1.792, *p* = 0.087); both perceived quality and perceived monetary savings positively influenced purchase intention. When perceived social status was low, the effect of perceived monetary savings was marginally significant (*b* = −0.344, *t* = −1.882, *p* = 0.070), and the effect of perceived quality was insignificant (*b* = 0.245, *t* = 1.416, *p* = 0.169); perceived monetary savings negatively influenced purchase intention.

### Event-Related Potential Results

**FIGURE 4:**
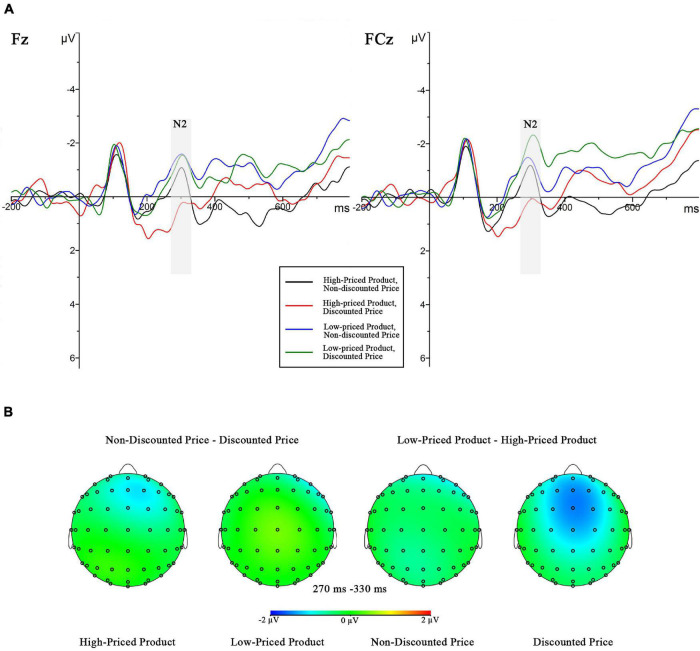
**(A)** Grand-averaged event-related potential (ERP) waveforms within the N2 time window (270–330 ms) across four conditions (high-priced product with discounted price or with non-discounted price, low-priced product with discounted price or with non-discounted price) with two electrodes (Fz and FCz). **(B)** Difference maps corresponding to the N2 amplitudes.

**FIGURE 5:**
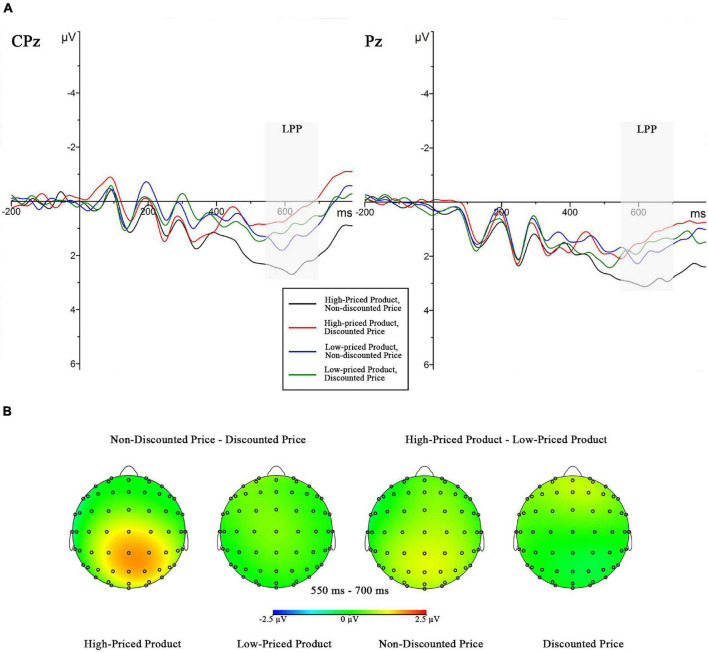
**(A)** Grand-averaged ERP waveforms within the late positive potential (LPP) time window (550–700 ms) across four conditions (high-priced product with discounted price or with non-discounted price, low-priced product with discounted price or with non-discounted price) with two electrodes (CPz and Pz). **(B)** Difference maps corresponding to the LPP amplitudes.

The grand mean ERPs and the brain topographies are shown in Figures 4, 5. The three-way 2 (original price: high vs. low) × 2 (price promotion: present vs. absent) × 10 (electrode) repeated-measure ANOVAs for N2 and LPP were conducted. The ANOVA of N2 revealed a marginally significant main effect on the original price [*F*_(1,18)_ = 3.492, *p* = 0.078, η${}_{\text{p}}^{2}$ = 0.162] and a significant effect on the price promotion [*F*_(1,18)_ = 6.017, *p* = 0.025, η${}_{\text{p}}^{2}$ = 0.251]. The interaction effect between the two conditions was marginally significant [*F*_(1,18)_ = 3.116, *p* = 0.094, η${}_{\text{p}}^{2}$ = 0.148]. The simple effect analyses were conducted, which showed that when the original price was high, the N2 amplitude was more negative for the non-discounted price (−0.426 ± 0.527 μV) than the discounted price (0.414 ± 0.692 μV, *p* = 0.021) and that when the original price was low, the significant difference between the non-discounted (−0.805 ± 0.447 μV) and discounted prices (−0.684 ± 0.450 μV, *p* = 0.595) was not found.

The LPP result showed two significant main effects of the original price [*F*_(1,18)_ = 5.422, *p* = 0.032, η${}_{\text{p}}^{2}$ = 0.231] and the price promotion [*F*_(1,18)_ = 5.681, *p* = 0.028, η${}_{\text{p}}^{2}$ = 0.240] as well as the marginally significant interaction effect between the two factors [*F*_(1,18)_ = 3.281, *p* = 0.087, η${}_{\text{p}}^{2}$ = 0.154]. As a confirmatory follow-up, the simple effect analyses revealed that in the high original price condition, the non-discounted price information evoked a higher LPP amplitude (2.522 ± 0.653 μV) than did the price promotion information (1.146 ± 0.608 μV, *p* = 0.010); in the low original price condition, the significant difference between the non-discounted (1.503 ± 0.642 μV) and discounted prices (1.337 ± 0.426 μV, *p* = 0.718) was not observed.

Because P2 and N400 amplitudes seemed different among four conditions in the frontal electrodes, we also conducted ANOVAs for these two components. The 160- to 220-ms time window was selected for P2, and the 380–440 ms was selected for N400. Ten electrodes (F3, F1, Fz, F2, F4, FC3, FC1, FCz, FC2, and FC4) were specified for P2 and N400. The results on P2 and N400 amplitudes showed that all the main and interaction effects were insignificant (*Fs <* 2.55, *ps* > 0.12).

## Discussion

The main purpose of the current study was to investigate price promotions’ effects on purchasing affordable luxury products at different original prices and the neural mechanism underlying these behaviors. Although affordable luxuries are similar to traditional luxuries in many aspects, such as high quality and high social status, price promotions, as a common marketing strategy, do not cause consistent influences on these two types of luxury. Since price exclusivity can be regarded as a method of meeting the need for social status, the influences of price promotions on the high-priced and the low-priced affordable luxuries could be differential. In addition, to better understand the effectiveness of price promotions, we applied the ERP technique to give us a measure of the moment-by-moment brain activity to reveal the cognitive processing of different price information.

Behaviorally, higher purchase intention was found in the high-priced affordable luxury product with price promotions than in the high-priced affordable luxury product with non-discounted price, whereas the difference in purchase intension was not significant in the low-priced condition. The following rating task showed that consumers perceived higher quality, higher social status, and less perceived monetary savings in the high-priced product compared with the low-priced, as well as in the non-discounted price than the discounted price. Furthermore, the original price moderated the influence of perceived monetary savings on purchase intention to affect the effectiveness of price promotions. The moderation effect of original price was mediated by perceived social status: When perceived social status was high, both perceived quality and perceived monetary savings had positive influence on purchase intention; when perceived social status was low, perceived monetary savings had negative influence on purchase intention. Combining these results from the formal experiment and the post questionnaire, we contend that consumers need to weigh up three factors product quality, social status, and monetary savings when making a purchase decision. When affordable luxuries are high priced, consumers perceived high social status. In this condition, price promotions positively influence affordable luxury purchases and perceived monetary savings, and price promotions negatively affect perceived quality; meantime, perceived monetary savings and perceived quality positively promote purchases; by weighing up these relationships, consumers have a stronger purchase intention toward price promotions. When affordable luxuries are low priced, consumers perceived low social status. In this condition, price promotions positively influence affordable luxury purchases and perceived monetary savings, and price promotions negatively affect perceived quality; meantime, perceived monetary savings negatively promote purchases; by weighing up these relationships, consumers would not show preference to price promotions. In addition, we observed a significant effect of the price promotion in the reaction time for the low-priced affordable luxury product. Previous studies have suggested that the reaction time is positively associated with task difficulty and cognitive load (Sweller, 1988; Wang et al., 2016). A shorter reaction time was found in the price promotion condition than in the absent price promotion condition. It takes less cognitive effort for consumers to decide whether to buy a low-priced product with a price promotion. Although consumers display low purchase intention to a low-priced product no matter whether it is with a price discount or not, they could make it more likely to form a negative attitude toward a discounted price.

Regarding the ERP component, the amplitude of N2 may be indicative of cognitive conflict (Folstein and Van Petten, 2008). The conflict will emerge when expectation stimuli are not presented to participants in a body of consumer neuroscience research (e.g., Mei et al., 2021). In the current study, we obtained a marginally significant interaction effect between the original price and the price promotion, and the N2 amplitude was less negative in the high-priced affordable luxury item with a price promotion than in the high-priced affordable luxury item without a price promotion, but it is not the case in the low original price condition. Consumers expect high quality and status granting from affordable luxuries at relatively low price (Mundel et al., 2017). As the post questionnaire shows, consumers can perceive a high-priced affordable luxury product as high quality and high social status and price promotions can create savings of paying for affordable luxuries. By weighing up these factors, consumers exhibit a more positive attitude and stronger purchase intention to purchase a high-priced product with a price discount, which belongs to a kind of expectation stimuli. Meantime, though a high original price lives up to the expectation of consumers, a non-discounted current price cannot help to acquire the benefits of affordable luxuries at a relatively low price. Similarly, a low-priced affordable luxury product with a price discount saves much more money, but this product’s quality and social status produced by this product to signal are too low to reach the expectation of consumers. Since consumers anticipate a high-priced affordable luxury item with a price discount, the presentation of a non-discounted current price produces the conflict between the presented information and the expected information and evokes an enhanced N2 amplitude. Meantime, there will be no significant discrepancy between non-discounted and discounted current prices in perceived conflict and N2 amplitude.

Late positive potential is a late positive-going component that is sensitive to the evaluative properties of stimuli and incongruence with a preceding context (e.g., Dhont et al., 2012). When people are asked to evaluate stimuli (positive or negative), the LPP amplitude is influenced by preceding stimuli, and it is larger to evaluative inconsistent stimuli (positive prime and negative target, or negative prime and positive target) than evaluative consistent stimuli (positive prime and positive target, or negative prime and negative target). In the current study, we found a more positive LPP amplitude in non-discounted price condition than in discounted price condition when the original price was high, and there was no significant difference when the original price was low. Consumers regard high-priced affordable luxuries as the products of high quality and signaling social status, and thus, high original prices are positive information as context stimuli or prime stimuli. As consumers are less willing to purchase an affordable luxury product without a price promotion than with one, they would form a negative attitude when a non-discounted price is presented, which is evaluatively incongruent with the preceding stimuli (i.e., positive–negative), leading to an increase in LPP amplitude than the discounted price stimuli preceded by high original price stimuli (i.e., positive–positive). Besides, a low-priced affordable luxury item is perceived to a decline in quality and to have difficulty in social granting; thus, low original price is a kind of negative information, whereas consumers show low purchase intention to a low-priced product regardless of with a price discount or without. When a current price preceded by low original price is non-discounted or discounted, consumers would evaluate both prices as negative information, which is evaluatively consistent conditions (i.e., negative-negative) and leads to insignificant difference in LPP amplitude.

On the other hand, practical implications for sellers should be discussed. First, we found that price promotions are effective in promoting the purchases of high-priced affordable luxuries, and that for this type of luxuries, consumers expect price promotion and give a positive evaluation. Sellers should take part in some shopping festivals, such as Double 11 in China, Black Friday in America, which can attract many consumers, to promote high-priced affordable luxuries to increase sales. Second, the current results showed that price promotions are inappropriate for promoting the purchases of low-priced affordable luxuries. Consumers perceive low product quality and low social status for low-priced affordable luxuries regardless of whether price promotions are present or absent. Thus, sellers are capable of designing promotional advertisement that show consumers what social status low-priced affordable luxuries can represent and how low-priced affordable luxuries are of good quality.

## Limitations and Extentions

First, during the current experiment, the evaluatively consistent condition evoked a higher LPP amplitude than the evaluatively inconsistent condition. In the research of Jing et al. (2019), a hedonic product picture was first presented. Subsequently, subjects were exposed to promotion information and made purchase decision. Though hedonic products, which can meet emotional needs and improve living standards, are regarded as the positive context stimuli, positive information (i.e., positive–positive, an evaluative consistent condition) elicits a larger LPP amplitude than negative information (i.e., positive–negative, an evaluative inconsistent condition) (Jing et al., 2019). In this regard, one could speculate that consumers could have focused on promotion information but paid less attention to hedonic pictures, whereas subjects need to know original prices (context stimuli) before forming attitudes in the current study. Future research should explore whether conscious and unconscious signals can modulate the amplitude deflection of the LPP component in the consumer neuroscience area.

Second, some factors could influence the effectiveness of price promotions. In the experiment, only female consumers were recruited for the preselected affordable brand, but the gender plays a significant role in moderating brand perception and purchase intention (e.g., Gilal et al., 2018, 2020); Yang et al. (2015) showed that consumers who are high in need for status exhibit a negative attitude toward a luxury hotel with a price promotion. It seems that need for status plays a key role in doing promotions; the effectiveness of price promotions has been found in utilitarian and hedonic items as well (Kivetz and Zheng, 2017), but the discount level increases promotional effectiveness for utilitarian more than for hedonic products (Eisenbeiss et al., 2015); participants could have speculated that the affordable luxuries with a high price were genuine and that the ones with a low price were counterfeit, since counterfeit products are generally cheaper than genuine products (e.g., Wu and Chiu, 2014). If the official price did not emerge, participants might have served low-priced products as normal ones. In sum, gender, need for status, discount levels, and whether the official price is concealed or revealed are all valuable extensions in the future.

Third, price promotion is a common type of marketing strategy in regular merchandise. However, since affordable luxury products have many similar aspects to traditional luxuries as mentioned in the *Introduction* section, many marketing strategies as effective method in promoting traditional luxury purchases like behavioral targeting (Yu et al., 2017), cross-market selling channel strategies (Huang et al., 2020), and social media marketing activities (Athwal et al., 2019) should be investigated further in affordable luxuries. Also, future research can focus on the affordable luxuries and traditional luxury purchases together to explore the effects of price promotions or other factors mentioned above. At last, it is noted that there were some marginally significant effects in behavioral and ERP results. One of the reasons might be that the sample size is too low in the study. Future research could use a greater sample size to further verify these findings.

## Conclusion

Behaviorally, price promotions for a high-priced affordable luxury product are effective, but it is not the case for a low-priced affordable luxury product, which is the result of weighing up product quality, social status, and monetary savings. Consumers respond more quickly to make decisions to discounted prices than non-discounted prices when original prices are low because of lower processing difficulty. At the neural level, people expect a high original price and a discounted price, and thus, other price information produces conflict and elicits a more negative N2 amplitude. The LPP amplitude is larger to a high-priced affordable luxury without a price discount than a high-priced affordable luxury with a price discount. The LPP amplitude differences are the result of the evaluative inconsistency effect, and the former is an evaluative inconsistent condition, and the latter is an evaluative consistent condition.

## Data Availability Statement

The raw data supporting the conclusions of this article will be made available by the authors, without undue reservation.

## Ethics Statement

The studies involving human participants were reviewed and approved by the Internal Review Board of the Laboratory of Cognitive Neuroscience, Yanshan University. The patients/participants provided their written informed consent to participate in this study.

## Author Contributions

KJ, LC, and YM conceived and designed the experiment. KJ and LC performed the experiment, wrote, and edited the manuscript. KJ and YM analyzed the data. All authors contributed to the article and approved the submitted version.

## Conflict of Interest

The authors declare that the research was conducted in the absence of any commercial or financial relationships that could be construed as a potential conflict of interest.

## Publisher’s Note

All claims expressed in this article are solely those of the authors and do not necessarily represent those of their affiliated organizations, or those of the publisher, the editors and the reviewers. Any product that may be evaluated in this article, or claim that may be made by its manufacturer, is not guaranteed or endorsed by the publisher.

## Footnotes

1 Since the original price influenced perceived quality, we also considered perceived quality as a mediator for analysis.

## References

[B1] Ajitha S. Sivakumar V. J. (2019). The moderating role of age and gender on the attitude towards new luxury fashion brands. *J. Fashion Mark. Manag.* 23 440–465. 10.1108/JFMM-05-2018-0074

[B2] Athwal N. Istanbulluoglu D. McCormack S. E. (2019). The allure of luxury brands’ social media activities: a uses and gratifications perspective. *Inf. Technol. People* 32 603–626. 10.1108/ITP-01-2018-0017

[B3] Baron M. Kenny A. (1986). The moderator-mediator variable distinction in social psychological research: conceptual, strategic, and statistical considerations. *J. Pers. Soc. Psychol.* 51 1173–1182. 3806354 10.1037//0022-3514.51.6.1173

[B4] Bellezza S. Keinan A. (2014). Brand tourists: how non-core users enhance the brand image by eliciting pride. *J. Consum. Res.* 41 397–417. 10.1086/676679

[B5] Brainard D. H. (1997). The psychophysics toolbox. *Spat. Vis.* 10 433–436. 10.1163/156856897X00357 9176952

[B6] Bruin K. J. Wijers A. A. (2002). Inhibition, response mode, and stimulus probability: a comparative event-related potential study. *Clin. Neurophysiol.* 113 1172–1182. 10.1016/S1388-2457(02)00141-4 12088714

[B7] Cacioppo J. T. Crites S. L. Berntson G. G. Coles M. G. H. (1993). If attitudes affect how stimuli are processed, should they not affect the event-related brain potential? *Psychol. Sci.* 4 108–112. 10.1111/j.1467-9280.1993.tb00470.x

[B8] Chandon P. Wansink B. Laurent G. (2000). A benefit congruency framework of sales promotion effectiveness. *J. Mark.* 64 65–81. 10.1509/jmkg.64.4.65.18071 11670861

[B9] Chen M. Ma Q. Li M. Dai S. Wang X. Shu L. (2010). The neural and psychological basis of herding in purchasing books online: an event-related potential study. *Cyberpsychol. Behav. Soc. Netw.* 13 321–328. 10.1089/cyber.2009.0142 20557253

[B10] Correia A. Kozak M. Kim S. (2018). Luxury shopping orientations of mainland Chinese tourists in Hong Kong: their shopping destination. *Tour. Econ.* 24 92–108. 10.1177/1354816617725453

[B11] Dhont K. Van Hiel A. Pattyn S. Onraet E. Severens E. (2012). A step into the anarchist’s mind: examining political attitudes and ideology through event-related brain potentials. *Soc. Cogn. Affect. Neurosci.* 7 296–303. 10.1093/scan/nsr009 21421734 PMC3304480

[B12] Dodds W. B. Monroe K. B. Grewal D. (1991). Effects of price, brand, and store information on buyers’ product evaluations. *J. Mark. Res*. 28 307–319. 10.1177/002224379102800305

[B13] Eisenbeiss M. Wilken R. Skiera B. Cornelissen M. (2015). What makes deal-of-the-day promotions really effective? The interplay of discount and time constraint with product type. *Int. J. Res. Mark.* 32 387–397. 10.1016/j.ijresmar.2015.05.007

[B14] Folstein J. R. Van Petten C. (2008). Influence of cognitive control and mismatch on the N2 component of the ERP: a review. *Psychophysiology* 45 152–171. 10.1111/j.1469-8986.2007.00602.x 17850238 PMC2365910

[B15] Gilal F. G. Zhang J. Gilal N. G. Gilal R. G. (2018). Integrating self-determined needs into the relationship among product design, willingness-to-pay a premium, and word-of-mouth: a cross-cultural gender-specific study. *Psychol. Res. Behav. Manag.* 11 227–241. 10.2147/PRBM.S161269 29922102 PMC5996856

[B16] Gilal F. G. Zhang J. Gilal R. G. Gilal N. G. (2020). Linking motivational regulation to brand passion in a moderated model of customer gender and age: an organismic integration theory perspective. *Rev. Manag. Sci.* 14 87–113. 10.1007/s11846-018-0287-y

[B17] Hajcak G. Olvet D. M. (2008). The persistence of attention to emotion: brain potentials during and after picture presentation. *Emotion* 8 250–255. 10.1037/1528-3542.8.2.250 18410198

[B18] Han Y. J. Nunes J. C. Drèze X. (2010). Signaling status with luxury goods: the role of brand prominence. *J. Mark.* 74 15–30. 10.1509/jmkg.74.4.15 11670861

[B19] Herring D. R. Taylor J. H. White K. R. Crites S. L. (2011). Electrophysiological responses to evaluative priming: the LPP is sensitive to incongruity. *Emotion* 11 794–806. 10.1037/a0022804 21517156

[B20] Huang H. He Y. Chen J. (2020). Cross-market selling channel strategies in an international luxury Brand’s supply chain with gray markets. *Trans. Res. Part E Logist. Trans. Rev.* 144:102157. 10.1016/j.tre.2020.102157

[B21] Huang J. Yuan Y. (2017). Bayesian dynamic mediation analysis. *Psychol. Methods* 22 667–686. 10.1037/met0000073 27123750

[B22] Ito T. A. Larsen J. T. Smith N. K. Cacioppo J. T. (1998). Negative information weights more heavily on the brain: the negativity bias in evaluative categorizations. *J. Pers. Soc. Psychol.* 75 887–900. 10.1037/0022-3514.75.4.887 9825526

[B23] Jang S. Moutinho L. (2019). Do price promotions drive consumer spending on luxury hotel services? The moderating roles of room price and user-generated content. *Int. J. Hosp. Manag.* 78 27–35. 10.1016/j.ijhm.2018.11.010

[B24] Jin J. Zhang W. Chen M. (2017). How consumers are affected by product descriptions in online shopping: event-related potentials evidence of the attribute framing effect. *Neurosci. Res.* 125 21–28. 10.1016/j.neures.2017.07.006 28734975

[B25] Jing K. Mei Y. Song Z. Wang H. Shi R. (2019). How do price and quantity promotions affect hedonic purchases? An ERPs study. *Front. Neurosci.* 13:526. 10.3389/fnins.2019.00526 PMC6558398 31231177

[B26] Kenny A. Korchmaros D. Bolger N. (2003). Lower level mediation in multilevel models. *Psychol. Methods* 8 115–128. 10.1037/1082-989X.8.2.115 12924810

[B27] Kivetz R. Zheng Y. (2017). The effects of promotions on hedonic versus utilitarian purchases. *J. Consum. Psychol.* 27 59–68. 10.1016/j.jcps.2016.05.005

[B28] Mazzocco P. J. Rucker D. D. Galinsky A. D. Anderson E. T. (2012). Direct and vicarious conspicuous consumption: identification with low-status groups increases the desire for high-status goods. *J. Consum. Psychol.* 22 520–528. 10.1016/j.jcps.2012.07.002

[B29] Mei Y. Jing K. Chen L. Shi R. Song Z. (2021). An investigation of a frontal negative slow wave in a virtual hedonic purchase task. *Front. Hum. Neurosci.* 15:674312. 10.3389/fnhum.2021.674312 34248527 PMC8264297

[B30] Mundel J. Huddleston P. Vodermeier M. (2017). An exploratory study of consumers’ perceptions: what are affordable luxuries? *J. Retail. Consum. Serv.* 35 68–75. 10.1016/j.jretconser.2016.12.004

[B31] Shahid S. Islam J. U. Farooqi R. Thomas G. (2021). Affordable luxury consumption: an emerging market’s perspective. *Int. J. Res. Mark. Early Access* 10.1108/IJOEM-01-2021-0144

[B32] Shavitt S. Lowrey T. M. Han S. P. (1992). Attitude functions in advertising: the interactive role of products and self-monitoring. *J. Consum. Psychol.* 1 337–364. 10.1016/S1057-7408(08)80059-9

[B33] Silverstein M. Fiske N. (2005). *Trading Up: Why Consumers Want New Luxury Goods, and How Companies Create Them*, Revised Edn. New York, NY: Portfolio.

[B34] Sweller J. (1988). Cognitive load during problem solving: effects on learning. *Cogn. Sci.* 12 257–285. 10.1207/s15516709cog1202_4

[B35] Truong Y. McColl R. Kitchen J. P. (2009). New luxury brand positioning and the emergence of Masstige brands. *J. Brand Manag.* 16 375–382. 10.1057/bm.2009.1

[B36] Vigneron F. Johnson L. W. (2004). Measuring perceptions of brand luxury. *J. Brand Manag.* 11 484–506. 10.1057/palgrave.bm.2540194

[B37] Wang L. (2013). Luxury Sales to Exceed $318 Billion, Driven by Emerging Markets and ‘Affordable Luxury’. The Business of Fashion. Available online at: http://www.businessoffashion.com/2013/10/euromonitor-coach-michael-kors-louis-vuitton-versace-fflur-roberts.html> (accessed October 8, 2013).

[B38] Wang Q. Meng L. Liu M. Wang Q. Ma Q. (2016). How do social based cues influence consumers’ online purchase decisions? An event-related potential study. *Electron. Commer. Res.* 16 1–26. 10.1007/s10660-015-9209-0

[B39] Wang Y. Qiao F. (2020). The symbolic meaning of luxury-lite fashion brands among younger Chinese consumers. *J. Fashion Mark. Mang. Early Access* 24 83–98. 10.1108/JFMM-09-2019-0204

[B40] Wiedmann P. Hennigs N. Siebels A. (2009). Value-based segmentation of luxury consumption behavior. *Psychol. Mark.* 26 625–651. 10.1002/mar.20292

[B41] Wu C. W. Chiu H. H. (2014). Turning counterfeiting into advantage: the case of a durable good monopolist. *Appl. Econ. Lett.* 21 1122–1127. 10.1080/13504851.2014.912031

[B42] Yang W. Zhang L. Mattila A. S. (2015). Luxe for less: how do consumers react to luxury hotel price promotions? The moderating role of consumers’ need for status. *Cornell Hosp. Q.* 57 82–92. 10.1177/1938965515580133

[B43] Yu S. Hudders L. Cauberghe V. (2017). Targeting the luxury consumer a vice or virtue? A cross-cultural comparison of the effectiveness of behaviorally targeted ads. *J. Fashion Mark. Manag.* 21 187–205. 10.1108/JFMM-07-2016-0058

[B44] Zhang Q. Li X. Gold B. T. Jiang Y. (2010). Neural correlates of cross-domain affective priming. *Brain Res.* 1329 142–151. 10.1016/j.brainres.2010.03.021 20298681 PMC2857548

